# Characteristic CT Findings After Percutaneous Cryoablation Treatment of Malignant Lung Nodules

**DOI:** 10.1097/MD.0000000000001672

**Published:** 2015-10-23

**Authors:** Ammar Chaudhry, Vadim Grechushkin, Mahsa Hoshmand, Choo Won Kim, Andres Pena, Brett Huston, Yair Chaya, Thomas Bilfinger, William Moore

**Affiliations:** From the Stony Brook University Medical Center, 101 Nicolls Road, LVL 4, Stony Brook, NY.

## Abstract

Assess computed tomography (CT) imaging characteristics after percutaneous cryotherapy for lung cancer.

A retrospective IRB-approved analysis of 40 patients who underwent nonsurgical treatment for primary stage 1 lung cancer performed from January 2007 to March 2011 was included in this study. All procedures were performed using general anesthesia and CT guidance. Follow-up imaging with CT of the chest was obtained at 1 month, 3 months, 6 months, and 12 months postprocedure to evaluate the ablated lung nodule. Nodule surface area, density (in Hounsfield units), and presence or absence of cavitations were recorded. In addition, the degree of nodule enhancement was also recorded. Patients who were unable to obtain the aforementioned follow-up were excluded from the study.

Thirty-six patients underwent percutaneous cryoablation with men to women ratio of 75% with mean age for men 74.6 and mean age for women 74.3 years of age. The average nodule surface area preablation and postcryoablation at 1-, 3-, 6-, and 12-month follow-ups were 2.99, 7.86, 3.89, 3.18 and 3.07�cm^2^, respectively. The average precontrast nodule density before cryoablation was 8.9 and average precontrast nodule density postprocedure at 1, 3, 6, and 12 months follow-ups were 8.5, −5.9, −9.4, and −3.8 HU, respectively. There is increased attenuation of lung nodules over time with an average postcontrast enhancement of 11.4, 18.5, 16.1, and 25.7 HU at the aforementioned time intervals. Cavitations occurred in the cryoablation zone in 53% (19/36) of patients. 80.6% (29/36) of the cavitations in the cryoablation zone resolved within 12 months. Four patients (11%) had recurrence of tumor at the site of cryoablation and none of the patients had satellite or distant metastasis.

Our study shows that patients who underwent cryotherapy for lung nodules treatment had characteristic changes on follow-up CT including. The surface area of the nodule increases at the 1-month follow-up with subsequent gradual decrease in the surface area. Decreased nodule density (Hounsfield units) at each interval follow-up is associated with complete ablation of the lung cancer whereas increasing nodule density was suggestive of recurrence. Cavity formation within the region of the ablated nodule, most of which typically resolved within the first 3 to 6 months. Nodule enhancement is difficult to assess because of the limited data sets that are available.

## INTRODUCTION

Lung cancer is the most common cancer worldwide, with an estimated 1,600,000 new cases and 1,380,000 deaths in 2008.^1–3^ In the United States, there were estimated 226,160 new cases of lung cancer and 160,340 deaths in 2012, making it the leading cause of cancer-related death in both men and women.^1–3^ Surgical resection is the standard treatment approach for patients with early stage NSCLC and sometimes can also be performed in selected patients with a limited number of small metastases. Surgical selection is based upon many factors including age, pulmonary function, and comorbidities (such as cardiovascular, diabetes, end-stage renal disease, presence of additional malignancies, etc). Preoperative evaluation helps exclude patients at high risk for postoperative complications. Approximately 16% of all patients are deemed poor surgical candidates despite their early stage of disease. Of these patients, 30% are over the age of 75 years. The nonsurgical candidates are then usually recommended to undergo alternative treatments such as radiofrequency ablation, stereotactic body radiotherapy, external beam radiation, chemotherapy, and cryoablation. These interventions can help treat the neoplasm in efforts to either alleviate symptoms resulting from the tumor and/or in certain case may help improve survival by completely or partially eradicating the tumor.^1–4^

Cryoablation is a minimally invasive emerging treatment for malignant lung nodules in patients with Stage I nonsmall cell lung cancer (NSCLC), solitary metastases, and in select cases it can be performed on patients with a small number of pulmonary metastases. It is an alternative to patients in whom surgical resection is not an option due to advanced age or coexistent medical morbidities. The decision for cryoablation versus another nonsurgical modality is determined by a multidisciplinary team of a pulmonologist, medical oncologist, radiologist, thoracic surgeon, and a radiation oncologist. Every effort is made to identify those patients who will tolerate pulmonary resection as current evidence supports this to be the treatment of choice.^1,5–9^

Cryoablation of lung tumors is a relatively new application for this mode of therapy and very little is known about the expected postprocedural imaging findings that can be reliably used to accurately interpret evolution of the neoplasm postablation. In surgical resection, the absence of tumor along resection margins helps histopathologically determine successful treatment of the neoplasm. However, in ablation procedures, the tumor is left in-situ postablation and there is no histopathologic correlation available to verify complete and/or partial tumor ablation. Consequently, postprocedural imaging becomes the backbone of analyzing postablation tumor and assessing whether the therapy was effective, partially effective, or completely ineffective.^7–9^ As recurrence is always a potential in treated neoplasm, an accurate assessment is critical because early detection can allow for repeat procedure or alternative treatments to treat the residual and/or recurred tumor. Computed tomography and positron emission tomography with CT fusion are 2 modalities that can be used to assess the postablation zone in crosssection for response to the intervention as these are readily available in or around centers where oncologic treatment is provided. In this study we investigate CT appearance of lung cancers postcryoablation.

## MATERIALS AND METHODS

In this IRB-approved HIPPA compliant study, 40 patients who underwent nonsurgical treatment for primary stage 1 nonsmall cell lung cancer (NSCLC) performed from January 2007 to March 2011 were included in this study. All primary NSCLC were diagnosed by histopathologic evaluation from tissue specimen obtained via percutaneous or transbronchial lung biopsy. The patients were staged using whole-body positron emission tomography-computed tomography (PET-CT). All patient cases were reviewed by a tumor board comprised of pulmonologist, medical oncologist, radiologist, thoracic surgeon, and radiation oncologist. The tumor board decision to perform cryoablation was made on patients deemed inoperable using the American College of Surgeons Oncology Group (ACOSOG) National Institute of Health (NIH) criteria (see Table 1).^5^ All procedures were performed using general anesthesia and CT guidance; thus patients who could not tolerate general anesthesia were excluded from the study. Follow-up imaging with CT of the chest was obtained at 1 month, 3 months, 6 months, and 12 months postprocedure to evaluate the ablated lung nodule. Patients with at least 1 year of follow-up were included in the study. Patient with more than single tumor in the target lobe or who had received prior radiation to the malignant lung neoplasms were excluded from the study. Patients with tumor size >3 cm, or tumor invading the mediastinal structures, chest wall, segmental bronchi, vessels, or nerves were excluded from the study. Patients with concurrent metastatic disease to the lung from nonprimary NSCLC were also excluded from the study. Thirty-six patients were identified who underwent cryoablation and met the aforementioned inclusion criteria. All of these 36 patients were deemed poor surgical candidates due to advanced age along with other comorbid factors, including advanced emphysema, diabetes mellitus, and advanced coronary artery disease.

**TABLE 1:**
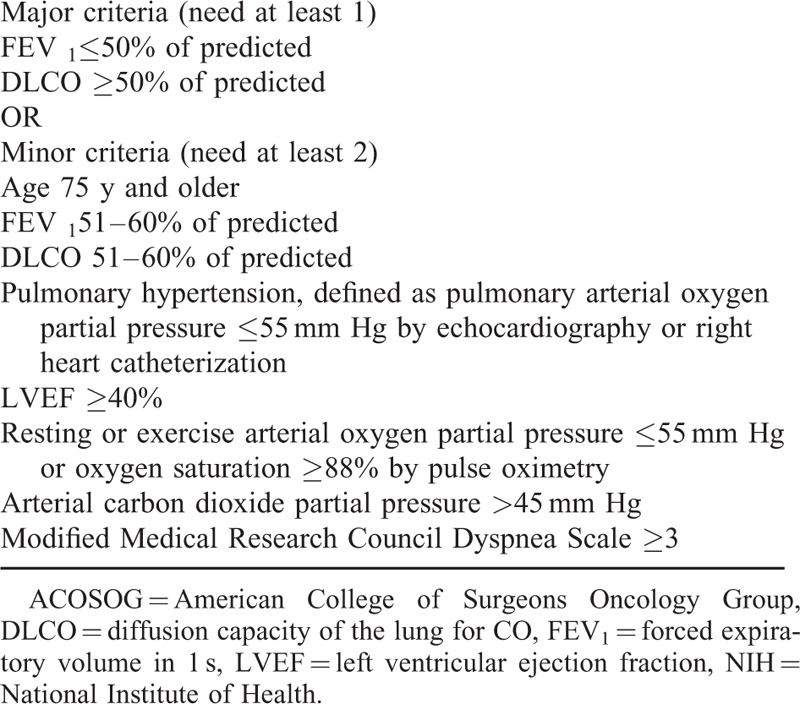
American College of Surgeons Oncology Group (ACOSOG) National Institute of Health (NIH) Criteria

## IMAGING STUDY PARAMETERS

There is no standard imaging protocol for postcryoablation follow-up published in the literature. Chest radiographs are routinely performed to evaluate for postprocedure complications immediately and within a week postcryoablation, which include pneumothorax and pulmonary hemorrhage. For follow-up of the ablation zone, CT is most commonly used as it is readily available and it is the standard of care for evaluation of lung cancer.^1^

### Noncontrast CT

All imaging was performed at the same institution using a General Electric (Milwaukee, WI) volume CT scanner. The scanner parameters were as follows: 64 × 0.625 detector configuration and pitch of 0.984:1. Images are displayed at 5 mm slice thickness in soft tissue window, 5 mm slice thickness in a lung algorithm, and 1.25 mm images also at lung algorithm. All images were interpreted on a 3 megapixel flat-panel color display, Barco medical system (Duluth, GA). The CT was used to localize the tumor and then later was used to confirm appropriate placement of the cryotherapy probe. Images were interpreted using both soft tissue (350 window and 50 level) and lung (1500 window and -600 level) algorithms. The imaging studies were performed from the base of the neck to the level of the inferior most aspect of the poster costophrenic angle. The unenhanced lung nodule density was measured using 3.00 mm region of interest (ROI) placed on the most dense region of the lung nodule. The lung nodule surface area was measured using the largest 2 dimensions as seen on axial, coronal, and/or sagittal plane. Unehanced CT examination was also used to evaluate for the presence or absence of cavitation in the cryoablation zone and was also used to measure the cavitations surface area using the largest 2 dimensions seen on axial, coronal, and/or sagittal plane.

### Contrast Enhanced CT

Swensen and colleagues demonstrated in a multicenter study that using nodule densitometry with a threshold increased enhancement of 15 Hounsfield units (HU) on postcontrast images, CT has detection sensitivity of ∼98%.^6^ Suh et al showed utility of CT densitometry in evaluate malignant pulmonary nodules postradiofrequency ablation with reported diminution of mean postcontrast enhancement at 1- to 2-month follow-up and marginally increased postcontrast enhancement at 3-month follow-up but less enhancing than the initial study.^11^ Abtin and colleagues used dynamic contrast-enhanced CT to evaluate malignant neoplasms postradiofrequency ablation by obtaining images at 45, 90, 180, and 300 s after administration of ∼100 mL of intravenous contrast at a rate of 2 to 3 mL per second.^12^ Abtin et al recorded nodule enhancement by subtracting precontrast malignant nodule attenuation from the postcontrast maximum nodule attenuation.^12,13^ As there is no published literature on evaluation of cryoablation zones in malignant neoplasms, we used a protocol similar to that used by Suh et al^11^ and Abtin et al.^12^

In our study, an average total of 110 mL of 300 concentration Ultravist (Bayer healthcare, Leverkusen, Germany) was administered. After initial noncontrast images, a CT of the entire chest from the base of the neck to the posterior most aspect of the costophrenic angle was performed at 30�s after the injection of contrast. Selective subsequent sequential additional CT scans were performed including only the cryoablation zones at 60, 120, 180, and 240�s time intervals.

### PET and PET/CT

In patients with lung cancer, PET and PET/CT have been considered by some^14–16^ as the standard for staging, surveillance of recurrent and metastatic disease, and in evaluation of treatment response to chemotherapy and radiation. Herrera and colleagues reported utility of PET in evaluating and confirming the presence of residual disease in the ablation zone. Higaki et al reported modest sensitivity (77.8%) and specificity (85.7%–90.5%) in predicting recurrence when evaluating patients’ postradiofrequency ablation at 1.5-, 3- to 6-, and 6- to 9-month intervals.^17^ The modest sensitivity and specificity along limited insurance coverage on serial PET-CT was not used for serial evaluation of residual and/or recurrence due to it modest sensitivity and specificity of PET-CT as well as limited insurance coverage of serial PET-CT.

### Image Analysis

The size of the ablation zone was measured in 2 dimensions on each imaging study. The postablation zone was characterized in the region of patients’ malignant pulmonary nodule by ground-glass opacity, which may partially or circumferentially envelope the initial lesion plus surrounding intra-lesional air-locules. Abtin et al^12^ and Goldberg et al^18^ had used similar criteria for defining postablation zones where the ground-glass opacities histologically represent the region of coagulative necrosis. The surface area (lesion length multiplied by lesion width) was recorded for each of the ablation zone. The presence or the absence of cavitations was recorded at each time point using 1 mm reconstructed images to determine the presence or the absence of cavitations in the ablation zone. Cavitation is defined as development of a “gas-filled spaced”^19^ within the cryoablation zone. The attenuation of the ablation zone was measured on precontrast images. Five separate measurements were obtained using a 5 millimeter^2^ region of interest (ROI) drawn at the central portion within each of the ablation zones taking care to avoid including cavitation as part of the measurement. These 5 measurements with an average together determine the overall density of the nodule. When contrast enhancement was available, the precontrast attenuation was evaluated using the same methodology as above and then, at each time point, the attenuation of the nodule was also measured. The maximum change in density was recorded.

### Cryoablation Protocol

The procedure was performed under general anesthesia. The target lung neoplasm and prospective cryoablation zone were identified on scout images performed on 16-slice General Electrics (Milwaukee, WI) helical CT Scan. Skin was cleansed with betadine after which 1% subcutaneous lidocaine was administered from skin to the pleura. All procedures utilized a percutaneous cryoablation device utilizing 2.4 mm or 3.0 mm diameter cryoprobe (CryoCare cryosurgical unit; Endocare, HealthTronics Irvine, CA). The number and configuration of the ablation devices were determined by the operator at the time of the procedure. After cleansing the skin, a 21-gauge guide needle was inserted along the planned optimized route using scout CT images evaluated in soft tissue, bone, and lung windows. The needle position was confirmed with repeat CT of the region of interest, after which a stainless steel cryoablation probe was inserted consisting of an external sheath and inner-guide needle. After confirming the position of the external sheath and inner-guide needle, the guide needle was removed and cryoprobe was introduced through the external sheath. The 3.0 mm cryoprobe was used for tumors sized >1.5 cm, and 2.4 mm cryoprobe was used for tumors sized <1.5 cm. Multiple probes were used for tumors sized >3.0 cm to ensure the adequate cryoablation zone. Multiple staged freeze-thaw-freeze cycle was performed on all tumors. The cycle began with a 10-min freeze period reaching temperature of at least −100°C followed by an 8-min decrease cycle raising the temperature to at least +20°C. In addition, a second cycle of repeat 10 min freezing followed by 8 min of thawing was performed reaching the minimum aforementioned temperatures. Following this, a third (last freeze cycle) was performed after which the cryoprobe was removed. The final set of images of the entire thorax was acquired to document the presence and/or absence of potential complications including pneumothorax, hemorrhage, and so on. Patients were initially evaluated postprocedure in the holding area and observed overnight. If no complications occurred, the patients were discharged home the subsequent morning with scheduled follow-up appointments.

### Follow-up

All patients in the study underwent follow-up CT thorax immediately following completion of cryoablation. After discharge from hospital, patients had a follow-up clinic appointment in 1-week to ensure no acute or subacute complications including chest pain, dyspnea, and so on, occurred. If patients were asymptomatic, they were scheduled for follow-up CT thorax 3-weeks from the clinic appointment, which was 4 weeks after the cryoablation. If asymptomatic and no acute findings noted on the follow-up CT thorax, patients were scheduled to obtain additional follow-up CT exams at 3 months, 6 months, and 12 months postprocedure to evaluate the cryoablation zone using the aforementioned protocol.

## RESULTS

**FIGURE 1:**
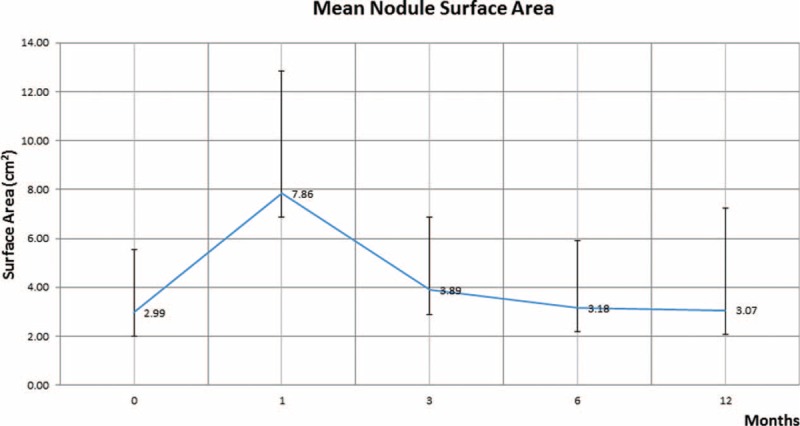
Shows the mean nodule surface area changed over time from a preablation means size of 2.99 cm^2^ to ∼7.86 cm^2^ at 1 month and then gradually decreased to a mean size of 3.07 cm^2^ at 12 months.

A total of 36 patients were included in this study, 27 males and 9 females (men to women ratio was 3:1). The mean age for men was 74.6 years of age and the mean age for women was 74.3 years of age. One of the most common findings in the immediate postprocedural time period after cryoablation is alveolar hemorrhage, which on imaging appears as ground glass opacities in and around the cryoablation. It was noted in 97.2% (35/36) patients and this finding typically resolved by 3 months in 97.1% (34/35) of the patients. The remaining 2.9% (1/35) patients had these ground glass opacities resolve by 6 months. The mean nodule surface area increased over time from preablation of 2.99 cm^2^ (±2.54 SD) to 7.86 cm^2^ (±4.99 SD) at 1-month interval (Figure 1). Paired *t* test analysis reveals mean difference of −4.88 (95% CI −6.389 to −3.366) with *P* < 0.0001, which means there is a statistically significant difference between the paired observations in these 2 groups. The mean nodule surface area at a 3-month follow-up interval was noted to have decreased to 3.89 cm^2^ (±3.00 SD). Paired *t* test analysis for difference in the surface area between 1 and 3-month follow-up revealed a mean difference between the groups of 3.95 (95% CI: 2.984–4.966) with *P* < 0.0001, which is statistically significant. The mean nodule surface area at 6-month follow-up interval was 3.18 cm^2^ (±2.73 SD). Paired *t* test analysis for the difference between the nodule surface area between 3- and 6-month follow-up revealed mean group difference of 0.708 (95% CI 0.119–1.298) with a *P* value of 0.0200, which is also statistically significant. At 12-month follow-up interval, the mean nodule surface area was 3.07 cm^2^ (±4.17 SD). Paired *t* test analysis revealed group mean difference of 0.108 (95% CI: −1.309 to 1.525) with a *P* value of 0.878, which is not statistically significant.

**FIGURE 2:**
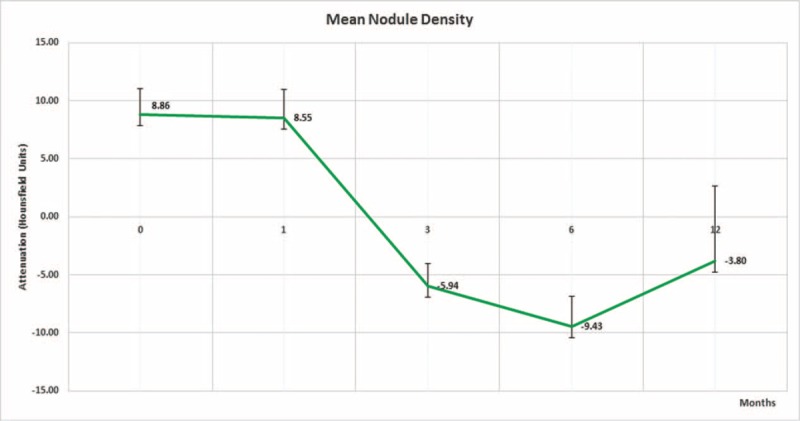
Shows the mean noncontrast attenuation of each of the lung nodules over time. Before cryoablation the mean noncontrast density of the lung cancer was 8.9 HU. At 1 month the mean nodular density decreased to 8.5 HU finally increasing to a mean value of −3.8 HU at 12 months.

The mean lung nodule density as measured on noncontrast CT (HU) precryoablation using the aforementioned technique revealed average nodule density of 8.9 (±2.19) HU, which had a slight decrease in attenuation at 1-month follow-up of −0.4 HU to an average of 8.5 (±2.46) HU. Paired *t* test analysis revealed mean group difference of 0.3125 (95% CI: −20.894 to 21.519) with *P* value of 0.976, which was not statistically significant. There was a more substantial decrease in nodule attenuation at 3- to 6-month follow-up of -3.0 HU and -18.2 HU to an average of 5.9 (±1.89)HU and -9.4 (±2.58)HU, respectively (Figure 2). The paired *t* test revealed mean group difference between 1- and 3-month follow-up and 3- to 6-month follow-up of 14.493 (95% CI: 10.206–39.192) and 3.484 (95% CI: −8.360 to 15.328) with *P* value of 0.2416 and 0.554, respectively. However, these differences were not statistically significant. Average density of the cryoablation zone at 12 months was −3.8 (±6.47), HU which is an approximate decrease of −12.7 HU. The paired *t* test revealed the mean group difference of 12.049 (95% CI: 0.175–23.923) between 6- and 12-month follow-up nodule attenuation with *P* value of 0.047, which was statistically significant. One patient was found to have recurrence at 6-month follow-up interval, which was confirmed by CT-guided biopsy of the lesion. The recurred neoplasm in the cryoablation zone demonstrates an increase in attenuation on noncontrast CT examination of +11.1 HU measuring 20 HU. At 12-month, 3 additional patients were found to have tumor recurrence at this time. The CT attenuation of the cryoablation zone in these patients increased from baseline average attenuation of 8.9 HU to 44 HU with a mean increase of +35 HU. Due to the small sample size of recurrence, statistically significant comparative analysis between recurrence and nonrecurrence groups could not be performed. On postcontrast CT, the lung nodules demonstrated evidence of postcontrast enhancement as evidenced by an overall increase in the attenuation of the lung nodules. Similar to image analysis of precontrast CT images of the cryoablation zone, on postcontrast images, 5 separate measurements were obtained using a 5 mm^2^ ROI drawn at the central portion within each of the cryoablation zones taking care to avoid including cavitation as part of the measurement. The values were then averaged and recorded to represent mean nodule enhancement on postcontrast scans. The mean preprocedural nodule attenuation was 21.5 (±3.72) HU. The mean cryoablation zone attenuation after contrast administration was 11.4 (±4.61) HU at 1-month follow-up and paired *t* test analysis revealed the mean group difference between precryoablation and 1-month follow-up nodule attenuation difference of 10.1 (95% CI: −21.998 to 1.798) with *P* value of <0.0001, which is statistically significant. At 3-month follow-up, the mean cryoablation zone attenuation was 18.5 (±2.38) HU and paired *t* test revealed the mean group difference between 1-month and 3-month follow-up of −7.1 (95% CI: −9.144 to −5.056) with *P* value of <0.0001, which is statistically significant. At 6-month follow-up, the mean postcontrast cryoablation zone attenuation was 16.1 (±5.29) HU. The paired *t* test revealed a mean group difference between 3-month and 6-month follow-up of 2.4 (95% CI: 0.115–4.685) with *P* value of 0.0399, which is statistically significant. At 12-month follow-up, mean postcontrast attenuation was 25.7(±4.83) HU and the paired *t* test revealed the mean group difference between 6- and 12-month follow-up of 9.6 (95% CI: −12.422 to −6.77) with *P* value of <0.0001, which was also statistically significant. Note, in 10 of the 36 patients intravenous contrast was contraindicated either secondary to renal function or allergies to intravenous contrast.

**FIGURE 3:**
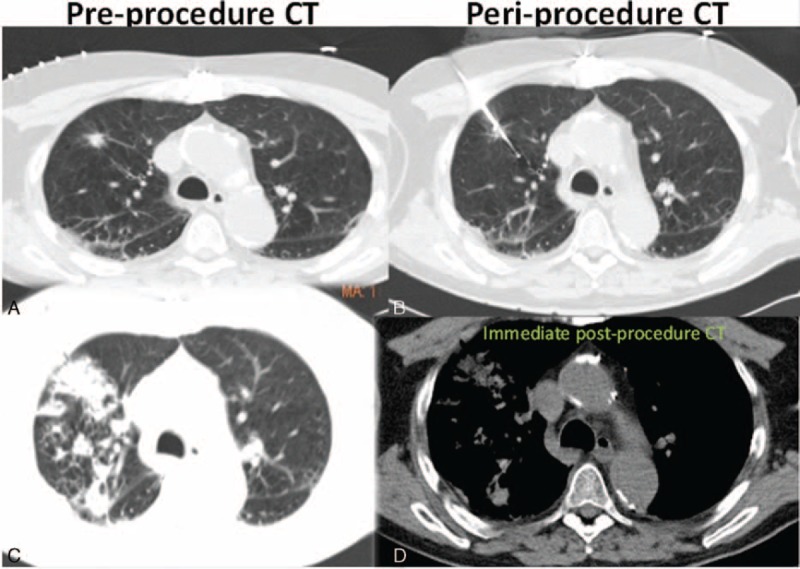
Shows a typical patient status postcryoablation procedure. The precontrast axial CT. A shows a biopsy proven adenocarcinoma in the right upper lobe. B shows the percutaneous cryoablation device centered in the lung cancer. C and D both show the postprocedural hemorrhage which is typically seen in cryotherapy. Please note that a freeze-thaw-freeze cycle was performed on all these patients for continuity of care and not a “triple freeze” protocol. CT = computed tomography.

**FIGURE 4:**
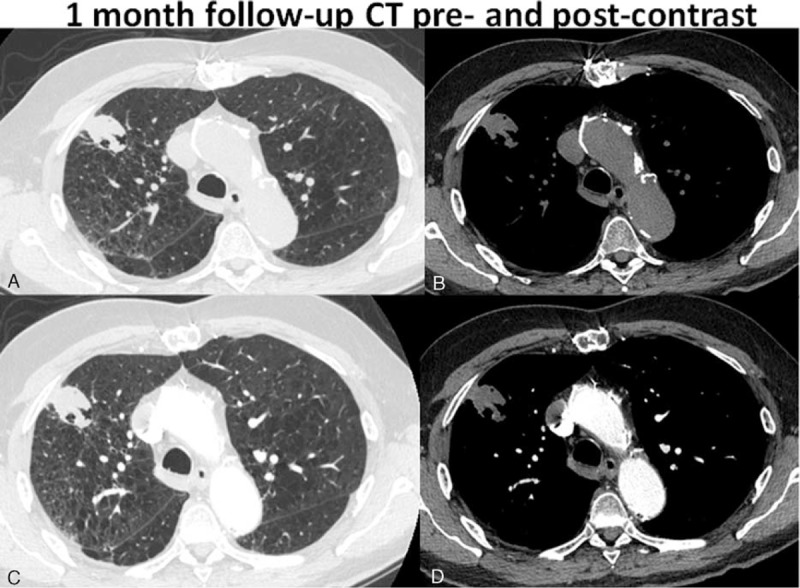
Shows the typical changes seen postcryoablation at 1-month follow-up. A shows cavitation medially in the cryoablation zone and overall the surface area of the cryoablation zone has increased in size compared to the preablation lung nodule. B shows the axial precontrast image in soft tissue window revealing relatively homogeneous density similar to that of muscle. C and D are postcontrast images in lung and soft tissue algorithm. Note that the attenuation of the nodule (D) has not changed significantly on postcontrast images as compared to precontrast images, which according to our data suggests that there is no evidence of residual/recurrent malignancy.

**FIGURE 5:**
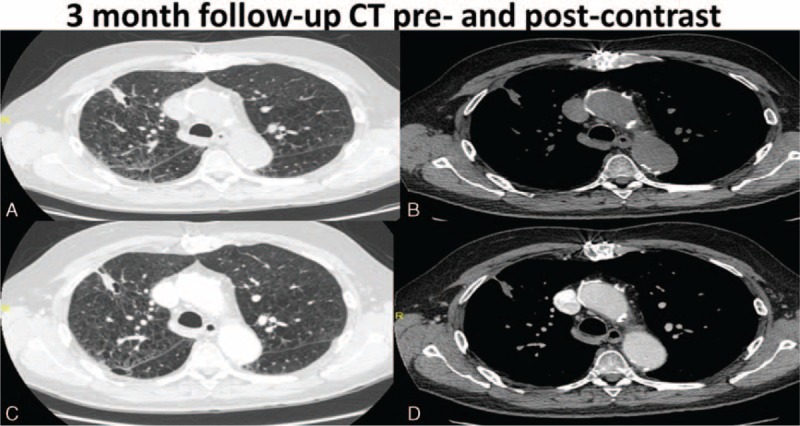
Shows the typical changes seen 3-month postcryoablation. A and B axial precontrast in both long and soft tissue windows show a relatively homogeneous dense cryoablation zone with density similar to that of muscle with interval decrease in overall cryoablation zone size and cavitation size. C and D axial postcontrast images in lung and soft tissue algorithm show that the density of the nodule has not changed significantly compared to precontrast images suggesting no evidence of residual/recurrent malignancy.

**FIGURE 6:**
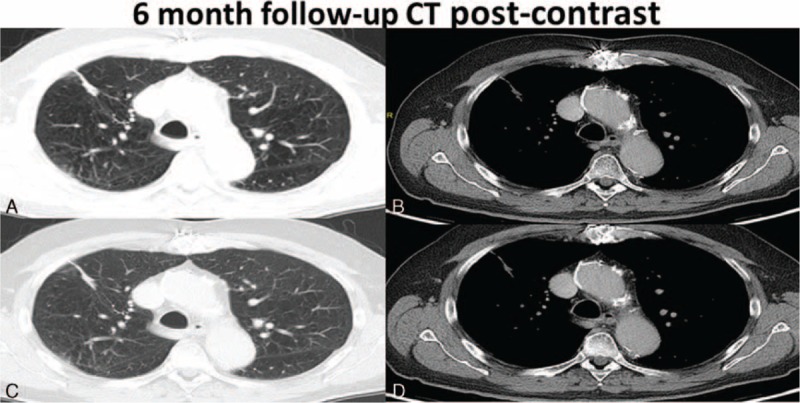
Shows the typical changes seen 6-month postcryoablation. A to D axial postcontrast in both long and soft tissue windows show continued decrease in size of the cryoablation zone, interval resolution in cavitation, and the density of the nodule has not changed significantly compared to precontrast images suggesting no evidence of residual/recurrent malignancy.

**FIGURE 7:**
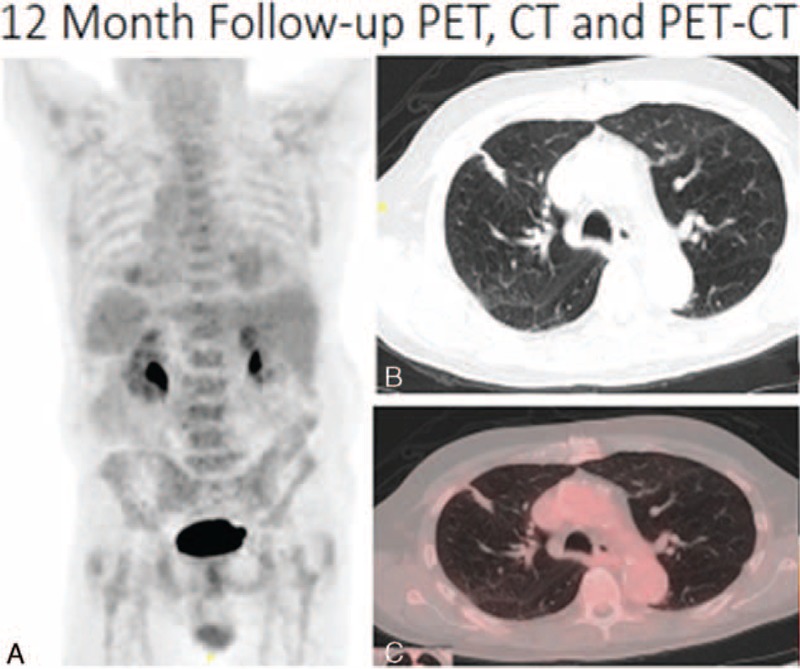
Reveals PET, CT, and PET-CT findings at 12-month follow-up. A (whole body PET) does not demonstrate evidence FDG uptake in the cryoablation zone where preablation was evidence of radiotracer activity. B and C reveal residual scar-like opacity in the right upper lobe cryoablation zone without interval increase in CT attenuation of the cryoablation zone.

Cavitation was present typically at the 1-month postablation scan and was seen in a total of 53% (19/36) of the patients. Most of these cavitations, 89.5% (17/19) resolved by the 12-month follow-up scan. Specifically, 73.7% (14/19) cavitations resolved at 3-month follow-up, 84.2% (16/19) cavitations resolved at 6 months, and 10.5% (2/19) cavitations had not resolved at 12 months. Interestingly, none of the patients with the presence of cavitation postcryoablation developed recurrence. These findings are evident in Figures 3 to 7, which demonstrate the typical pre- and postcryoablation imaging findings along with typical CT findings seen at 1-, 3-, 6-, and 12-month follow-up in a 76-year old men with newly diagnosed stage I NSCLC. Figure 3 shows a typical patient pre- (Figure 3A), peri- (Figure 3B), and immediately postcryoablation procedure (Figure 3C and D). The precontrast axial CT Figure 3A shows a biopsy proven adenocarcinoma in the right upper lobe, which was PET-. Figure 3B shows the percutaneous cryoablation device centered in the lung cancer. Figure 3C and D both show the postprocedural hemorrhage, which is typically seen in cryotherapy. Please note that the freeze-thaw-freeze cycle was performed on all of these patients for continuity of care and not a “triple freeze” protocol. Figure 4 shows the typical changes seen postcryoablation at 1-month follow-up. Figure 4A shows cavitation medially in the cryoablation zone and overall the surface area of the cryoablation zone has increased in size compared to the preablation lung nodule. Figure 4B shows the axial precontrast image in soft tissue window revealing relatively homogeneous density similar to that of muscle. Figure 4C and D are postcontrast images in lung and soft tissue algorithm. Note that the attenuation of the nodule (Figure 4D) has not changed significantly on postcontrast images as compared to precontrast images, which according to our data suggests that there is no evidence of residual/recurrent malignancy. Figure 5 shows the typical changes seen 3-month postcryoablation. Figure 5A and B axial precontrast in both long and soft tissue windows shows a relatively homogeneous dense cryoablation zone with density similar to that of muscle with interval decrease in overall cryoablation zone size and cavitation size. Figure 5C and D axial postcontrast images in lung and soft tissue algorithm shows that the density of the nodule has not changed significantly compared to precontrast images suggesting no evidence of residual/recurrent malignancy. Figure 6 shows the typical changes seen 6-month postcryoablation. Figure 6A to D axial postcontrast in both long and soft tissue windows shows continued decrease in size of the cryoablation zone, interval resolution in cavitation, and the density of the nodule has not changed significantly compared to precontrast images suggesting no evidence of residual/recurrent malignancy. Figure 7A whole body PET does not demonstrate evidence of FDG uptake in the cryoablation zone where preablation was evidence of radiotracer activity. Figure 7B and C reveals residual scar-like opacity in the right upper lobe cryoablation zone without interval increase in CT attenuation of the cryoablation zone.

## DISCUSSION

According to American College of Chest Physicians (ACCP), lobectomy is considered the standard of care for stage I NSCLC.^20,21^ Earlier versions of the guidelines were more dismissive of the minimally invasive therapies such as RFA, cryoablation, and so on, due to paucity of data.^20^ However, the more recent guidelines from ACCP have acknowledged the utility and efficacy of minimally invasive therapies and are recommending them especially in patients with comorbidities.^21^ Lung cancer is the third most common cause of cancer in United States; however, it is the most common cause of cancer-associated death in men and women.^22^ Approximately 224,230 new cases of lung cancers were diagnosed in 2013 and ∼159,260 patients had lung cancer-related death in United States alone.^23^ Globally, ∼1.4 million deaths were attributed lung cancer.^23^ As patients with lung cancer tend to be older in age (30% are older than age 75), occasionally have various comorbidities, and are deemed poor surgical candidate (∼16%), there is an expected rise in the application of minimally invasive therapies.^1–5,24,25^

As such, familiarity with the expected benign postprocedural findings and the ability to discriminate them from recurrence is critically important. Our study shows characteristic pattern changes associated with treatment of lung cancer with cryotherapy and will assist clinical radiologists in evaluating expected changes after lung cancer cryoablation (see Table 2). In patients with early stage lung cancers status postcryoablation, there is an early increase in the surface area, which peaks around 1 month status postcryoablation followed by a gradual decrease in the surface area. The surface area increases at 1 month secondary to the mobilization of macrophages and neutrophils into the cryoablation zone as part of the postcryoablation immunologic cascade, which leads to tumor lysis and ensuing tissue repair.^26^ Subsequently, as concentration of cellular debris, macrophages, and neutrophils decreases, there is steady reduction in the size of the cryoablation zone as noted on follow-up CT in our study.^26^ More than 50% of the patients showed cavitation early after cryotherapy in the ablation zone. The cavitation typically resolved over time typically at the 3- to 6-month follow-up CT scans. The patients who developed cavitations did not develop recurrence for uncertain reasons. One reason could be that cryoablation in these patients lead to significant coagulative necrosis yielding rapid cell death and dissolution with interim replacement of tumor cells with air cavity, which gradually collapsed upon development of scar tissue over time.^26^

**TABLE 2:**
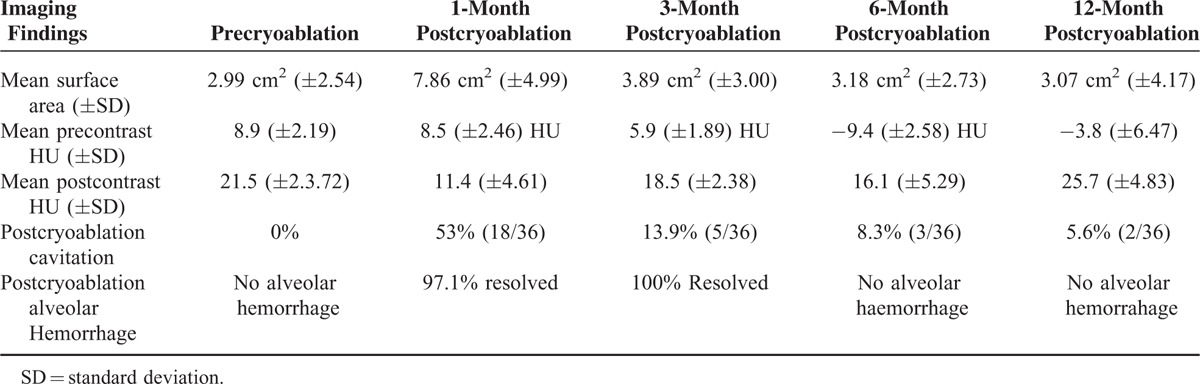
Characteristic Postcryoablation Imaging Findings

Our study also shows that on unenhanced CT examination, the cryoablation zone density (measured in HU) was noted to decrease over time and decreased nodule density at each interval follow-up is associated with complete ablation of the lung cancer. In our study, all patients with biopsy proven recurrence of lung cancer had the density of the cryoablation zone increase between follow-up intervals. One patient was found to have recurrence at 6-month follow-up in whom the cryoablation zone demonstrated an interval increase of +11 HU, whereas 3 patients with recurrence at 12-month follow-up were noted to have an average increase in attenuation of +35 HU. The change in cryoablation zone attenuation after contrast administration in the nonrecurrence group showed a slight overall increase in the cryoablation zone over time, generally <10 HU at each of the follow-up intervals. These findings suggest successful treatment response from cryoablation if nodule attenuation on interval follow-up on postcontrast examination does not increase by >10 HU.

There are several limitations to the study. First, as this is a retrospective study, it suffers from disadvantages inherent to the study design including selection bias. It is also difficult to assess the temporal relationship as one cannot control for exposure and other variables in a retrospective study design. Another limitation of the study is in evaluation of postcontrast enhancement due to the inhomogeneous data from the nodule enhancement studies. There are a few missing time points as some patients were unable to have intravenous contrast due to acute or chronic renal insufficiency. However, when excluding patients who were known to have recurrent malignancy in the ablation zone, the pattern of enhancement does show an overall decrease enhancement compared to precryoablation enhancement. Additionally, the relatively small size of the study limits generalizability to all patients with cryotherapy especially patients with cryotherapy for nonprimary lung malignancies although, one would expect a similar pattern. There are prior studies which have been recently published on this topic. Ito et al evaluated 56 patients who had cryoablation in a total of 79 different tumors in the lung of which 12 were primary lung cancers and the remaining were metastatic disease from multiple different primary sources. In their article they showed similar increase in the overall nodules size early after cryoablation and first a rapid decrease in size and then a slower decrease in size. Our results are similar, although the early rapid decrease in size was not observed in our cohort of patient.^32^ Additionally, nodule enhancement data from other studies in the literature showed no significant relationship between the tumor recurrence and evidence of nodule enhancement on postcontrast images.^5,6,27–31^ In our study, 4 patients dramatically affected the overall mean attenuation on noncontrast CT as discussed in the *Results* section. Therefore, the presence of interval increased CT attenuation in the cryoablation zone on noncontrast CT may represent a more reliable indicator of recurrence. Interestingly, these patients also had relatively high absolute postcontrast enhancement numbers of 44 HU and 35 HU. The patient in this cohort with documented recurrence at 6 months was noted to have postcontrast attenuation of the cryoablation zone of 80 HU, which was a significant change from early studies. This may suggest that nodule enhancement may be beneficial in evaluating the postablation zone in primary lung cancer.

The presence of cavitation appears to be critically important as suggested from the radiofrequency ablation of literature.^4^ In this study, cavitation was present in 53% of the patients; this is greater than reported by Ito et al who in their cohort of patients found cavitation only 35% of their patients. However, Wang et al found cavitation in nearly 80% of their patients. However, both Ito et al and Wang et al performed cryotherapy on predominantly nonprimary lung carcinoma.^10,32^ None of the patients who were shown to later have tumor recur in the ablation zone had evidence of cavitation at any time during the follow-up period. This finding suggests that cavitation may be a surrogate marker for good clinical outcome as has been suggested in prior articles regarding repeat with the ablation.

In conclusion, with the rise in incidence and deaths secondary to lung cancer and an ever-growing complex patient population with multiple comorbidities, minimally invasive techniques (such as cryoablation) are becoming a key treatment option for patients with early stage NSCLC. As all these patients are followed clinically with CT, familiarity with benign and malignant imaging findings is crucial in patient management. Continuous decrease in the cryoablation zone size after 1-month follow-up, presence of cavitation, decreased CT attenuation of cryoablation zone, and increase in enhancement of cryoablation zones of <10 HU on interval follow-up are major findings of our study which signify the absence of recurrence. Due to our retrospective study design and small sample size, future studies with larger cohort of patients will be necessary to validate these findings before they can be generalized to a large population. Additionally, as our study focused solely on early stage NSCLC, additional multicenter studies are necessary to understand how early stage primary lung cancers differ from advanced staged primary lung cancers and/or metastatic cancers to the lung with respect to their response to cryotherapy and postprocedural imaging findings.
